# Insulin/Insulin-Like Growth Factors in Cancer: New Roles for the Aryl Hydrocarbon Receptor, Tumor Resistance Mechanisms, and New Blocking Strategies

**DOI:** 10.3389/fendo.2015.00012

**Published:** 2015-02-02

**Authors:** Travis B. Salisbury, Justin K. Tomblin

**Affiliations:** ^1^Department of Pharmacology, Physiology and Toxicology, Joan C. Edwards School of Medicine, Marshall University, Huntington, WV, USA

**Keywords:** IGF1R, IGF1, insulin, AHR, insulin receptor-A subtype, biased-agonism, MED-573, OSI-906

## Abstract

The insulin-like growth factor 1 receptor (IGF1R) and the insulin receptor (IR) are receptor tyrosine kinases that are expressed in cancer cells. The results of different studies indicate that tumor proliferation and survival is dependent on the IGF1R and IR, and that their inhibition leads to reductions in proliferation and increases in cell death. Molecular targeting therapies that have been used in solid tumors include anti-IGF1R antibodies, anti-IGF1/IGF2 antibodies, and small molecule inhibitors that suppress IGF1R and IR kinase activity. New advances in the molecular basis of anti-IGF1R blocking antibodies reveal they are biased agonists and promote the binding of IGF1 to integrin β3 receptors in some cancer cells. Our recent reports indicate that pharmacological aryl hydrocarbon receptor (AHR) ligands inhibit breast cancer cell responses to IGFs, suggesting that targeting AHR may have benefit in cancers whose proliferation and survival are dependent on insulin/IGF signaling. Novel aspects of IGF1R/IR in cancer, such as biased agonism, integrin β3 signaling, AHR, and new therapeutic targeting strategies will be discussed.

## A Short History of Insulin/IGFs in Cancer

### IGF1R

The early evidence linking the IGF1R to cancer was the finding that the transformation of mouse embryo fibroblasts (MEFs) by many, but not all, tested oncogenes requires an intact *Igf1r* gene. For instance, the SV40 large T antigen, *H-Ras*, *EWS/FLI-1*, and *c-Src* transform wild type, but not *Igf1r* null, MEFs (1–4). *G*_α_*_13_* and *v-Src* induces the transformation of wild type and *Igf1r* null MEFs (4, 5). Transgenic overexpression of oncogenic *Kras* in the murine mammary gland induces the formation of mammary tumors that overexpress *Igf1r* (6). Such tumors resemble human basal-like breast tumors that are resistant to therapy (6). The growth of *Kras* expressing murine mammary tumors is delayed upon deletion of the *Igf1r* gene from mammary tumors (6). Treating mice with the IGF1R inhibitor picropodophyllin (PPP) suppressed the growth of *Kras* expressing mammary tumors compared with vehicle (6). PPP also inhibited the growth of MDA-MB-231 breast cancer xenografts in mice (6). Collectively, these reports provided *in vitro* and *in vivo* evidence that the IGF1R promotes transformation and the progression of breast cancer.

### IGF1

Liver specific *Igf1* knockout mice have lower levels of circulating IGF1 (by ~75%) than wild type mice (7, 8). Lowering the levels of circulating IGF1 in mice has been shown to inhibit the growth of colon cancer xenografts and there is reduced incidence of metastatic spread to the liver (7). Additionally, exogenous IGF1 increases the growth and metastasis of colon cancer in mice (7). Similar results were observed in murine models of breast cancer. Specifically, breast tumors grow slower in IGF1 deficient mice than wild type mice (9). On the other hand, transgenic overexpression of the human *IGF1* gene in epithelial cells of the mouse prostate induces the formation of spontaneous prostate cancer (10). In humans, acromegaly is associated with higher incidence rates of colorectal cancer (11). In contrast, Laron-type dwarfism is associated with low IGF1 levels and reduced cancer risk (12). Thus, high levels of IGF1 are associated with increased incidence of cancer progression, while lower levels of IGF1 are associated with decreased incidence of cancer progression in mice and humans.

Canonical signaling responses to insulin/IGFs have been reviewed (13–16). Insulin/IGFs upon activation of their cognate receptors induce PI3K and MAPK signaling. Increases in PI3K and MAPK signaling in cancer cells induce proliferation and resistance to cell death (17, 18). In addition to the canonical insulin/IGF pathways, recent work indicates that insulin receptor substrate 1 (IRS-1) and the IGF1R translocate from the cell membrane into the nucleus in response to IGF1 (19, 20). In the nucleus, IRS-1 binds to the promoters of *CCND1* and *cMYC* (21). In doing so, IRS-1 increases the expression of *CCND1* and *cMYC* (21). These findings provided a mechanism by which IGF1 through IRS-1 increases proliferation because *CCND1* and *cMYC* induce cell cycle advance (21). IRS-1 also binds to the promoter of ribosomal DNA (21). The binding of IRS-1 to the ribosomal DNA promoter promotes ribosomal RNA synthesis, which is required for increases in cell size (22). Ligand-induced translocation of the IGF1R into nucleus requires the IGF1R to undergo SUMOylation at specific lysine residues (Lys^1025^, Lys^110^, and Lys^1120^ in the β subunit) (23). Upon entering the nucleus, SUMOylated IGF1R binds to lymphoid enhancer-binding factor 1 (*LEF1*) on Wnt target gene promoters like *CCND1* and *AXIN* (24). By this mechanism, the IGF1R increases *CCND1* and *AXIN* expression (24).

### Insulin

Mice that express a dominant negative IGF1R in skeletal muscle (MRK mice) are insulin resistant and exhibit hyperinsulinemia (25). MRK mice are not obese and they have mild hyperglycemia (25). Mouse breast cancer cells that express oncogenes form tumors when grafted into the mammary fat pad of mice. The growth of such tumors is increased in MRK mice compared with wild type mice (26). High levels of insulin activate the insulin receptor (IR), but not the IGF1R, in tumors in MRK mice (27). Mice treated with the insulin analog AspB10 develop larger mammary tumors than vehicle-dosed mice (27). The IR, but not the IGF1R, is activated in tumors in mice treated with AspB10 (27). Western blot analysis reveals that MRK mammary tumors exhibit higher levels of phosphorylated AKT and S6 ribosomal protein (*S6rp*) than mammary tumors in control mice (28). MRK mice dosed with pan-class I PI3K inhibitor NVP-BKM120 or the dual PI3K/mTOR inhibitor BEZ235 had smaller tumors than MRK mice treated with vehicle (28). PPP inhibits tumor growth in MRK mice without inducing significant metabolic toxicity (29). This PPP benefit was attributed to partial inhibition of the IGF1R and IR, as discussed by the authors (29).

## TCDD and the Aryl Hydrocarbon Receptor Impact IGF2 Signaling in Breast Cancer Cells

Obesity increases the risk for several cancers including breast cancer (30). We (and others) have shown that adipocyte conditioned medium (adipo-CM) stimulates the proliferation of human breast cancer cells more than fibroblast conditioned medium (fibro-CM) (31, 32). We identified that adipocytes secrete higher levels of IGF2 than fibroblasts (32). Adipo-CM-stimulated breast cancer cell proliferation was inhibited with anti-IGF2 blocking antibody (32). 2,3,7,8 tetrachlorodibenzo-*p*-dioxin (TCDD) is a lipophilic toxicant that inhibits estrogen signaling and disrupts interactions between CCND1, CDK4, and RB1 (33, 34). We found that TCDD inhibits adipo-CM- or IGF2-stimulated breast cancer cell proliferation and reduces the expression of *E2F1*, *CCND1*, *MYB*, *SRC*, *JAK2*, and *JUND1* compared with vehicle (32). Taken together, these data suggest that TCDD inhibits adipo-CM and IGF2 signaling in breast cancer cells by downregulating the expression of genes that are important for sustaining high rates of proliferation (32). We are currently investigating signaling mechanisms by which TCDD regulates gene expression in human breast cancer cells stimulated with adipokines or IGF2.

The aryl hydrocarbon receptor (*AHR*) is a ligand-activated transcription factor that is best known for mediating the toxic effects of TCDD (35). Our recent findings indicate that AHR responds to and mediates IGF2 signaling in MCF7 breast cancer cells (36). We found that IGF2-treated MCF7 cells have higher levels of AHR mRNA and protein than control cells (36). We noted that increases in AHR protein correlated with increases in *CCND1* expression in cells treated with IGF2 (36). Chromatin immunoprecipitation (ChIP) experiments revealed that the binding of AHR to the *CCND1* gene promoter was increased in IGF2-stimulated MCF7 cells compared with vehicle-treated controls (36). We then knocked down AHR with specific interfering RNA and found that reducing AHR levels inhibited IGF2-stimulated increases in *CCND1* mRNA and protein in MCF7 cells (36). Considering that *CCND1* promotes cell cycle, we asked whether knockdown of AHR inhibits IGF2-stimulated MCF7 proliferation (36). AHR knockdown MCF7 cells are indeed less responsive to IGF2-mediated increases in proliferation than controls (36). Collectively, these findings indicate that IGF2 induces signaling in MCF7 cells that promotes the association of the AHR with the *CCND1* gene promoter, which in turn increases proliferation (36).

## Tumor Resistance Mechanisms to IGF1R Blocking Therapy

Considering the roles of IGF/IGF1R in transformation, tumor growth, and resistance to cell death, anti-IGF1R antibodies were designed for cancer therapy (17, 18). Problems associated with the anti-IGF1R antibodies included adverse endocrine effects and limited effectiveness (17, 18). The limited effectiveness of anti-IGF1R antibodies has been attributed to tumor resistance (17, 18). Recent work has established that IGF1R blocking antibodies have biased-agonism activity toward the IGF1R (37). Further, blockade of the IGF1R with antibody can promote IGF1 signaling through the integrin β3 receptor in tumor cells (38). Biased agonism and the binding of IGF1 to the integrin β3 receptor are novel mechanisms of tumor resistance to anti-IGF1R antibodies that we will discuss below.

## Anti-IGF1R Antibodies are Biased Agonists

**Figure 1:**
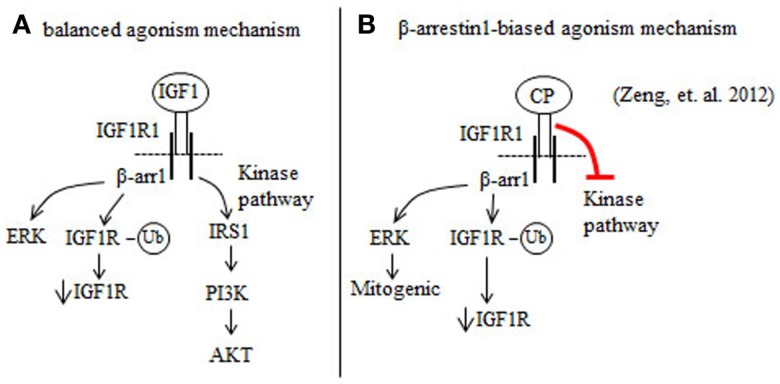
**Figitumumab (CP) is a biased agonist of the IGF1R that selectively promotes β-arrestin1 (β-arr1) signaling in cancer cells**. **(A)** IGF1 binding to and activation of the IGF1R stimulates the β-arr1 pathway that leads to proteasomal degradation of the IGF1R through a ubiquitin (Ub)-mediated mechanism and ERK activation. IGF1 binding to the IGF1R also induces IGF1R autophosphorylation and this serves to increase the kinase pathway that promotes the phosphorylation and activation of AKT. **(B)** CP binding to the IGF1R preferentially increases β-arr1 signaling, and inhibits the IGF1R kinase pathway. By this mechanism, CP downregulates IGF1R and AKT signaling, and increases ERK-mediated mitogenic signaling.

IGF1 is a balanced IGF1R agonist that induces beta arrestin 1 (β-arr1) and IGF1R kinase signaling pathways (39, 40) (Figure 1A). The anti-IGF1R antibody figitumumab (CP) is a biased IGF1R agonist because it suppresses IGF1R kinase activity, but activates β-arr1 signaling (37) (Figure 1B). Increases in β-arr1 signaling in response to CP will mediate mitogenic ERK signaling and proteasome-mediated downregulation of the IGF1R (37) (Figure 1B). Combining the ERK inhibitor UO126 with CP reduces the proliferation of Ewing’s sarcoma (ES) cells more than UO126 or CP alone (37). Thus, the results of Zheng et al. (37) indicate that blockade of ERK by ERK inhibitors may improve the clinical benefits of CP and other anti-IGF1R antibodies that are biased β-arr1 agonists (37).

## IGF1 Binds to Integrin β3

Shin and colleagues in 2013 tested the effectiveness of the anti-IGF1R antibody cixutumumab (cix) on a panel of human head and neck squamous cell carcinoma (HNSCC) and non-small cell lung cancer (NSCLC) cell lines (38). The authors found that the growth of some, but not all, tested cancer cell lines was inhibited by cix (38). Western blot analysis showed that the levels of phosphorylated Src, epidermal growth factor receptor (EGFR), AKT, mTOR, and p70S6K were higher in cix-treated resistant cancer cells than cix-sensitive cells (38). The authors recognized that IGF1 had previously been shown to bind to and activate integrin β3, but not integrin β1, on Chinese hamster ovary cells (41). Binding assays established that IGF1 also binds to integrin β3 on HNSCC cells (38). Inhibiting the binding of IGF1 to the integrin β3 receptor in cix-treated HNSCC cells blocked increases in Src signaling (38). Next, the authors transplanted HNSCC tumors from patients into mice (38). Such tumors were not growth inhibited in mice dosed with cix compared to controls (38). Knockdown of integrin β3 or inhibiting Src in primary human HNSCC rendered the tumor xenografts sensitive to cix treatment in mice (38). Collectively, these findings indicate that blockade of the IGF1R with cix induces IGF1 to bind to integrin β3, which in turn induces Src signaling that increases cancer cell growth (38).

## New Blocking Strategies

**Figure 2:**
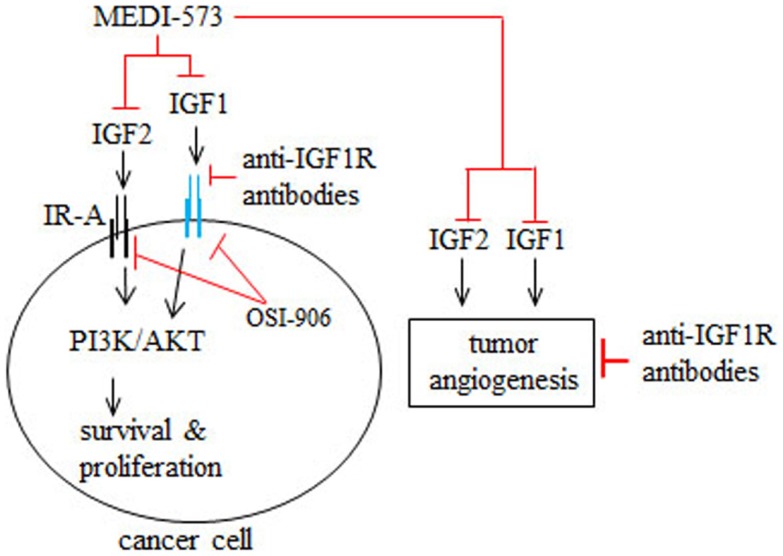
**Mechanism of action of MEDI-573, anti-IGF1R antibodies, and OSI-906 in cancer cells and tumor angiogenesis**. IGF2 binding to insulin receptor subtype A (IR-A) and IGF1 binding to IGF1R in cancer cells and tumor blood vessels promotes cancer cell survival and proliferation as well as tumor angiogenesis. MEDI-573 is a human antibody that neutralizes IGF2 and IGF1 and thus inhibits the cancer effects of both IGF ligands. Anti-IGF1R antibodies inhibit IGF1 signaling through IGF1R, but not IGF2 signaling mediated by IR-A. OSI-906 is a small molecule inhibitor that inhibits IGF1R and IR autophosphorylation and therefore IGF1 and IGF2 cancer effects.

MEDI-573 is a human antibody that selectively targets IGF1 and IGF2, but not insulin (42) (Figure 2). MEDI-573 affinity for human IGF2 is higher than its affinity for human IGF1 and its affinity for murine IGF1 is low (42). IGF2 binding to the insulin receptor isoform A (IR-A) in cancer cells has mitogenic and tumor promoting effects *in vitro* and *in vivo* (43, 44) (Figure 2). Because MEDI-573 targets IGF2, it could be particularly effective in tumors that overexpress IR-A (42). Combining an anti-IGF1R antibody with MEDI-573 offers a better antitumor effect because of greater inhibition of IGF1 and IGF2 signaling in cancer cells and tumor angiogenesis is inhibited (45) (Figure 2). In addition, MEDI-573 combined with an mTOR1 inhibitor (AZD2014) inhibits sarcoma xenografts more than MEDI-573 or mTOR1 inhibition alone (46). MEDI-573 has been tested in phase I clinical trials in solid tumors (47, 48). These trials showed that MEDI-573 effectively clears IGF1 and IGF2 from plasma in patients at doses below limiting toxicity (47, 48). The most frequent adverse effects of MEDI-573 were fatigue and gastrointestinal complaints (47, 48). Immunogenicity against MEDI-573 was evaluated and none was found (47, 48). Clinically, the tumor response to MEDI-573 was stable disease (in ~30% of patients) and no partial or complete responses occurred (47, 48).

OSI-906 is a small molecule IGF1R/IR kinase inhibitor that suppresses the growth of tumor xenografts in mice (49) (Figure 2). Phase I trials have tested intermittent versus continual dosing of OSI-906 in patients with advanced solid tumors (50, 51). In both OSI-906 trials, hyperglycemia was an adverse effect, which occurred more frequently in a diabetic cohort in the continual dosing study (51). Stable disease occurred in patients dosed intermittently or continually with OSI-906 (50, 51). Further, two patients with adrenocortical carcinoma had partial responses to intermittent doses of OSI-906 (50). In the continuous dose study, one patient with melanoma had a complete response to OSI-906 (51). Overall, OSI-906 was well tolerated has antitumor activity and warrants further study, as discussed by the authors (50, 51).

## Conclusion

From all apparent evidences, we propose that the insulin/IGF system is still an effective target for cancer therapy. There is still a need for uncovering new ways to effectively target both insulin and IGF signaling in cancer while avoiding significant metabolic toxicity. Part will come from the recognition of new pathways of tumor resistance. There is also a need to identify specific biomarkers to predict sensitivity or resistance to existing anti-IGF1R therapies (52). Thus, collectively, insulin/IGF signaling in cancer and its therapeutic targeting still warrants further investigation.

## Conflict of Interest Statement

The authors declare that the research was conducted in the absence of any commercial or financial relationships that could be construed as a potential conflict of interest.
